# Sero-prevalence and factors associated with anti-*Brucella* antibodies in slaughter livestock in Uganda

**DOI:** 10.3389/fepid.2023.1213592

**Published:** 2023-06-29

**Authors:** James Bugeza, Kristina Roesel, Ignacio Moriyon, Denis Mugizi, Lordrick Alinaitwe, Velma Kivali, Clovice Kankya, Elizabeth Anne Jessie Cook

**Affiliations:** ^1^ Department of Animal and Human Health, International Livestock Research Institute (ILRI), Kampala, Uganda; ^2^ Vaccinology Research Program, National Livestock Resources Research Institute (NaLIRRI), Kampala, Uganda; ^3^ College of Veterinary Medicine, Animal Resources and Biosecurity, Makerere University, Kampala, Uganda; ^4^ Department of Animal and Human Health, International Livestock Research Institute, Nairobi, Kenya; ^5^ Department of Microbiology and Parasitology, ISTUN (Instituto de Salud Tropical) y Depto. Microbiología y Parasitología Universidad de Navarra, Edificio de Investigación, Pamplona, España (Spain)

**Keywords:** antibodies, *Brucella*, *Brucellosis*, false positive serological reaction, livestock, sero-prevalence, Uganda

## Abstract

**Introduction:**

Brucellosis is endemic in Uganda and is a major cause of production losses in livestock. Early detection and quantification of the disease is vital for its control and eradication. The aim of this study was to assess the sero-prevalence and factors associated with anti-*Brucella* antibodies in slaughtered livestock.

**Materials and methods:**

Sera from 886 cattle, 925 small ruminants, and 900 pigs were collected from regional abattoirs in Northern, Eastern and Central Uganda. To estimate sero-prevalence, sera were serially tested using a combination of the Rose Bengal Test (RBT) and Native Hapten (NH) immunoprecipitation test. True sero-prevalence was estimated using the Rogan-Gladden estimator considering the sensitivity and specificity of the NH immunoprecipitation test. Multivariable logistic regression was used to assess factors associated with seropositivity for anti-*Brucella* antibodies.

**Results and discussion:**

Small ruminants showed the highest seroprevalence (6.7%, 95% CI = 4.2-7.1) followed by cattle (3.8%, 95% CI = 2.4-4.9) and pigs (2.8%, 95% CI = 1.1-2.9). Seropositivity for anti-*Brucella* antibodies was associated with region of origin (OR = 4.6,95%CI=1.49-17.75, *p* = 0.013) for cattle; sex (OR = 2.90, 95% C = 1.5-6.34, *p* = 0.004), age (OR=4.04, 95% CI = 1.07-8.52, *p* = 0.006) and species (OR = 2.53, 95% CI = 1.08-6.98, *p* = 0.048) for small ruminants; and finally sex for pigs (OR = 2.88, 95% CI = 1.07-8.52, *p* = 0.041). Progressive control interventions must include both cattle and small ruminants since they play a bigger role in the maintenance and dissemination of *Brucella*. The interventions should adopt a risk-based approach with regions at higher risk being given top priority. Bacteriological and molecular studies should be undertaken to clarify the role of pigs and the goat-cattle cross infections in the epidemiological cycle of brucellosis in Uganda.

## Introduction

Livestock, particularly cattle, small ruminants, and pigs, constitute major sources of much needed animal protein, but they are also a potential source of foodborne illnesses like brucellosis to consumers, industry workers and other vulnerable groups. Economic losses caused by this disease in livestock results from abortions, loss and or reduction in number of replacement stock, infertility, reduced milk yields and cost of disease management (1). Trade restrictions imposed on endemic countries also constitute another form of economic burden (2). Brucellosis remains a major constraint to livestock production and a threat to public health in endemic areas of Sub-Saharan Africa.

Brucellosis is caused by organisms belonging to the genus *Brucella* which have a characteristic host preference. Cattle, small ruminants and swine are the preferred hosts for *B. abortus*, *B. melitensis*, and *B. suis,* respectively, although cross species infections are common where mixed herding is practiced (3–5). *B. ovis*, a non-zoonotic rough *Brucella* species is restricted to sheep. Infection occurs through the oropharyngeal mucosa by ingestion of feed and water contaminated by secretions of aborted fetuses or by licking vaginal discharges, genitals, and new born calves of infected cows (6). Upon entry, *Brucella* cells are engulfed by phagocytes and transported to regional lymph nodes where they proliferate and thereafter disseminate via the blood stream and lymphatic route to localize in the reticuloendothelial system and the reproductive tract (7). In the new born and young animals, the *Brucella* species are extremely silent so that some are asymptomatic and serologically negative until the first parturition/ abortion when massive amounts of the organism are shed (8).

Because brucellosis lacks pathognomonic clinical signs, its diagnosis requires laboratory tests. Over the years several serological tests have been developed for detection and surveillance of brucellosis, and their proper use under different circumstances is essential to obtain a clear picture of the disease. Key among these tests is the Rose Bengal Test (RBT) which is recommended by the OIE for routine surveillance and control of brucellosis (9). Others include i-ELISA, c-ELISA, complement fixation (CFT), fluorescence polarization assay (FPA) and serum agglutination test (SAT). These assays rely on the detection of antibodies to the immunodominant C epitope of the smooth (S) lipopolysaccharide (S-LPS) (8). Two short comings of serological testing and interpretation of results in animal brucellosis are the false positive serological reactions (FPSR) caused by cross reacting bacteria (*Yersinia enterocolitica* O:9*, Escherichia coli* 0:157 and *Salmonella* group N) and the antibodies elicited after vaccination with S *Brucella* vaccines Rev1 and S19. Although RBT and i-ELISA show very high diagnostic sensitivity (DSe), they are affected by FPSR and, depending on the time after vaccination and age of the animals, are also affected by vaccination with S *Brucella* vaccines (9). There is a widely held misconception that the i-ELISA, c-ELISA and the FPA have the best diagnostic specificity (DSp) and can therefore be used as “confirmatory” tests after screening with RBT (10, 11). However, the c-ELISA does not resolve these problems and has comparatively less DSp (10). Much less information exists on the FPA, which is adversely affected by the conditions in resource-limited countries (12). On the other hand, the Native Hapten (NH) immunoprecipitation test shows only a small reduction in DSe as compared to RBT or i-ELISA, correlates with *Brucella* shedding, has excellent DSp in animals vaccinated with S *Brucella* and discriminates FPSR antibodies (9, 13).

Previous serological studies in Uganda reported high prevalence of *Brucella* antibodies in food animals particularly cattle and goats. For instance, Faye reported individual cattle prevalence of 15.8% using the RBT (14). Kabagambe found individual animal prevalence of 4% in goats using the card test (CT; essentially the same as RBT) (15). However, when they used the SAT the individual animal prevalence was 10% which is in contradiction with the lower DSe of SAT. In a more recent study Erume found that 2 out of 1,130 pig sera tested were positive using an i-ELISA but negative with CFT, suggesting that brucellosis is not a huge burden in these species (16). Since pigs are often infected by *Yersinia enterocolitica* O:9, these authors also investigated this infection. Using a SAT with *Y. enterocolitica* O:9 cells as antigen, they reported the existence of pigs infected with this bacterium suggesting FPSR for *Brucella*. However, this result needs to be verified because SAT positive results with either *Y. enterocolitica* O:9 or S *Brucella* antigens cannot discriminate brucellosis and *Y. enterocolitica* O:9 infections.

As seen from the above examples, prevalence estimates from past studies need to be interpreted carefully considering the characteristics of the tests and/or lack of validation for the local conditions (4, 10). This is also illustrated by the contradictory results of different i-ELISA kits and the SAT results with *Y. enterocolitica* O:9 (16). In addition, the use of RBT as a screening test and the CFT as “confirmatory” test should be done only when S19 and Rev1 vaccination is implemented, and retesting of tagged animals can be used to assess whether CFT titres decrease (vaccination) or increase (infection and anamnestic responses to *Brucellae* in the herd/flock). In addition the World Organization for Animal Health recommends CFT (or those other tests) as “complementary” (rather than “confirmatory”) because they can provide quantitative assessments of antibodies when successive retesting of suspicious animals or herds/flocks is conducted (9, 11). Therefore, further studies with careful application of serological testing and interpretation of results are needed.

Slaughterhouse surveillance is one of the approaches that can be used to monitor zoonotic diseases to guide the deployment of targeted interventions (17). In addition to being potential avenues for amplification and dissemination of *Brucella* species, slaughterhouses are collection points for livestock from several geographical areas and can serve as sentinel points for monitoring and surveillance. In this study we used this approach and a combination of the RBT and the NH immunoprecipitation test in a serial testing scheme to rule out FPSR and possible post-vaccination antibodies, thus detecting anti-*Brucella* antibodies due to natural infection with high specificity. Specifically, we examined the seroprevalence of anti-*Brucella* antibodies in 3 major animal food value chains namely cattle, small ruminants, and pigs at the point of slaughter.

## Materials and methods

### Study design

A cross sectional study design was adopted to collect animal samples and data from major slaughterhouses in 3 geographical regions of Uganda between December 2021 and December 2022.

### Study area

The study was conducted in 3 major regional slaughterhouses in the Central, Northern and Eastern parts of the country. In the Central region slaughterhouses were selected from Kampala, in the North from Lira and in the East from Mbale.

### Sample size determination and sample selection

A total of 886 cattle, 925 sheep and goats, and 900 swine sera samples determined using the Epitools-epidemiological calculator available at http://epitools.ausvet.com.au were collected. Sample sizes for each species per region were computed considering the sensitivity and specificity of the RBT, the assumed true prevalence, the precision, and confidence level. For this study the DSe and DSp of RBT were assumed to be 100%, a value slightly above those reported for cattle, sheep and goats (18, 19). The confidence level and precision were 0.95 and 0.05, respectively. Sero-prevalence of anti-*Brucella* antibodies from previous studies for each species of livestock were used for the above computations (15, 20–25). At the slaughter facility animals presented for slaughter were selected opportunistically. To avoid clustering, different species in each region were sampled on alternating days until the required number was obtained.

### Collection of blood samples and epidemiological data

Gushing blood was aseptically collected upon slaughter into a sterile wide mouth 50 ml plastic container. Approximately 10 ml of the blood was quickly transferred into paired gel separator tubes. The tubes were left to stand at room temperature and later packed on ice and dispatched to the Central Diagnostic Laboratory (CDL) at the College of Veterinary Medicine, Animal Resources and Biosecurity (CoVAB), Makerere University, Kampala. At CDL serum was aliquoted and stored at −20°C until testing was performed. Animal epidemiological data was collected and recorded.

### Testing of animal serum for anti-*Brucella* antibodies

Ruminant sera were screened for *Brucella* antibodies using the RBT, using the standard protocol (30 µl of serum plus 30 µl of antigen) for bovine and porcine sera and the modified protocol for caprine/ovine serum (i.e., 75 µl of serum plus 25 µl of antigen), as described in the OIE manual (9). The tests were performed at room temperature using a white tile onto which the serum and antigen mixture were placed and rocked gently for 4 min. Agglutination and non-agglutination denoted a positive and a negative reaction, respectively. The NH immunoprecipitation test was performed on all RBT positive samples using the agar gel immunodiffusion (AGID) protocol as previously described by Dieste-Perez (26)*.* The gel was made by adding 1 g of Noble Agar (BD Difco, Winnersh, UK) and 10 g of NaCl to 100 ml of borate buffer and dissolved by boiling under continuous stirring. Borate buffer was made by dissolving 12.4 g of boric acid (Merck Life Sciences, Dorset, UK) and 14.5 g of potassium chloride (Honeywell Specialty Chemicals Seelze GmbH, Hanover, Germany) in 1,600 ml of distilled water. An adequate amount of molten gel was poured on clean glass slides placed on a flat surface to form a bed of 2.5 mm (about 3.5 ml for standard slides). Once the gel was completely set (after about 15–20 min), holes of 5 mm diameter at 3 mm spacing were formed using a gel puncher forming hexagonal figures. A 5 mm diameter central hole was also made. Problem sera were placed in alternate wells separated by a positive control serum (infection proved by bacteriology), with the antigen in the central well. The antigen was comprised of 2 mg/ml of NH-rich S-LPS extract from *Brucella melitensis* 16M diluted with distilled water (27). The antigen was prepared from the Department of Microbiology and Parasitology, Universidad de Navarra, Pamplona, Spain. Briefly, the NH-rich preparation was obtained by heat extraction of *B. melitensis* 16M sequential ethanol precipitation, freeze dried and titrated against a panel of sera of *B. abortus* and *B. melitensis* infected ruminants (27). The slides were incubated for 24 and 48 h at room temperature in a humid chamber. Positive animals were denoted by double S-LPS and NH, or single NH precipitin lines (NH, closer to serum well and LPS, close to antigen well) implying that the animal was infected and most likely excreting *Brucella* organisms (8).

### Data analysis

For each species, apparent prevalence (AP) was calculated by dividing the number testing positive by the number tested (Number positive/Number tested) ×100. True prevalence (TP) was calculated using the formula adopted formula below as adopted from (28).

$$\mathrm{True}\;\mathrm{prevalence}=\frac{\mathrm{Apparent}\;\mathrm{prevalence}+\;(\mathrm{specificity}-1)}{\mathrm{Specificity}+\;(\mathrm{sensitivity}-1)}$$

The DSe and DSp of the NH immunoprecipitation test were found to be 92%–94% and 100% respectively in studies conducted in ruminants (19, 29), and 68% and 100% respectively in a study conducted by Dieste-Perez with pig sera (26). The relationship between anti-*Brucella* serostatus and the different epidemiological factors was assessed using Pearsons Chi-square test or Fishers exact test as appropriate. A multivariable logistic regression model was fitted using the glm function in R-studio version 4.2.2 to assess factors associated with anti-*Brucella* seropositivity. The Hosmer-Lemeshow (HL) goodness of fit test (in the *generalhoslem* package) with the backward selection procedure was used to select the best model. Odds Ratios (OR), 95% confidence interval (CI) and *p*-values were used to evaluate the association. Factors with *p*-values below or equal to 0.05 being considered significant. A map showing the districts of origin of slaughtered livestock was drawn using QGIS 3.30.1.

## Results

### Origin of slaughter livestock

**Figure 1:**
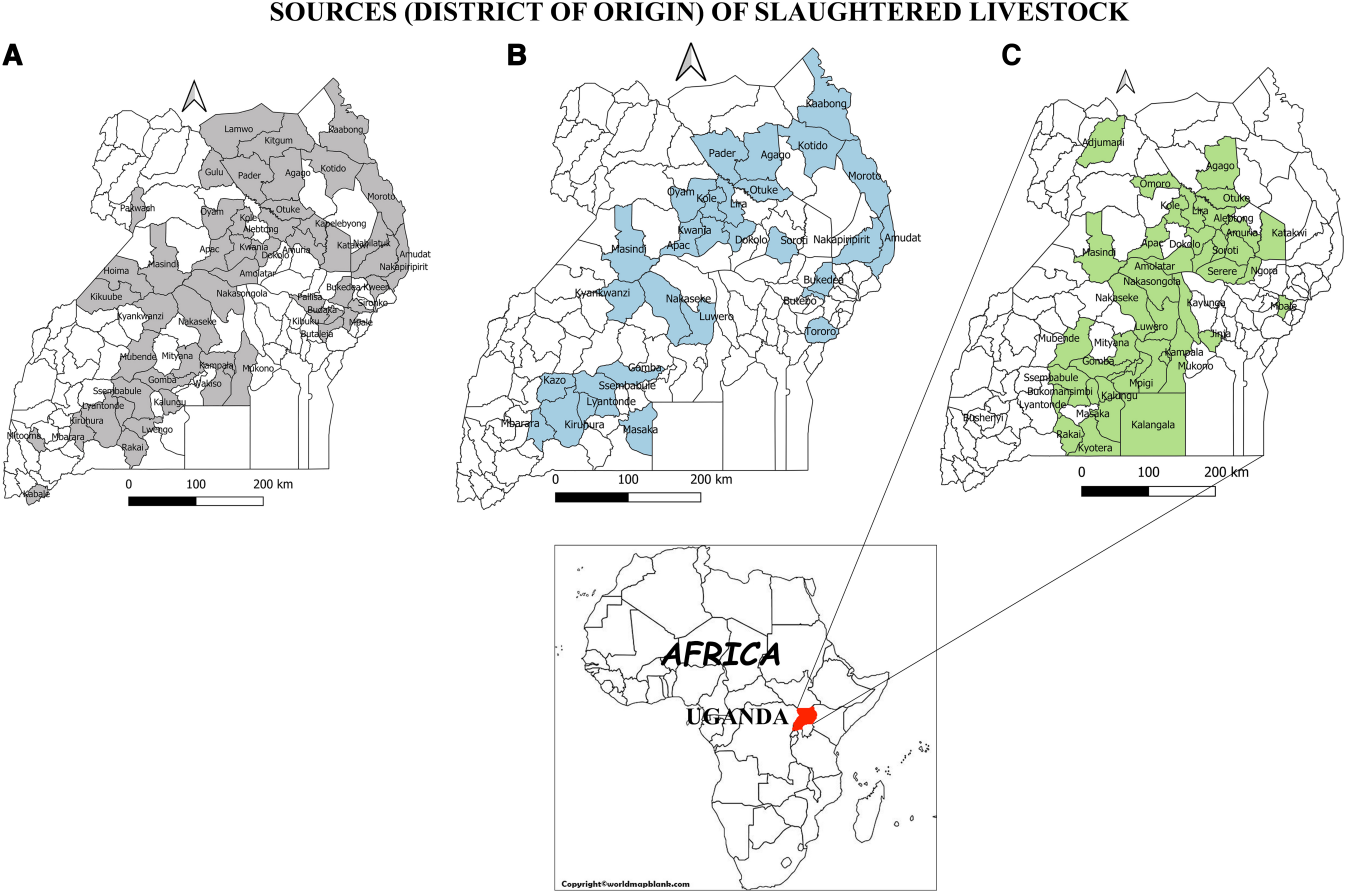
Map of Uganda indicating the districts of origin of slaughter livestock. (**A**) Origin of slaughtered cattle, (**B**) Origin of Slaughtered small ruminants, (**C**) slaughtered pigs.

The number of districts contributing both slaughter cattle and small ruminants were more (*n* = 48) (Figures 1A,B) compared to those contributing pigs (*n* = 43) (Figure 1C).

### Number of livestock sampled per region

In total, 886 cattle, and 925 small ruminants were sampled from Mbale City abattoir in the East of the country, Kampala city abattoir in the central part of the country and Lira City abattoir in the North of the country (Table 1). A total of 900 pigs were sampled from swine slaughter slabs in the North and Eastern parts of the country and from Wambizi abattoir in Kampala. Majority of cattle (45.3%,401/886) were sampled from Kampala city abattoir. Similarly, majority of small ruminants (38.5%, 356/925) and pigs (65.4%, 589/900) were sampled from Kampala (Table 1).

**Table 1:** Number of animals of each species sampled per region.

Region	Species (%)		
	Cattle	Small ruminants	Pigs
East	228 (25.7)	244 (26.4)	101 (11.2)
Central	401 (45.3)	356 (38.5)	589 (65.4
Northern	257 (29.0)	325 (35.1)	210 (23.3)
Total	886 (100)	925 (100)	900 (100)

### Seroprevalence in cattle, goats and sheep, and pigs

The highest prevalence (6.7%) of anti-*Brucella* antibodies was seen in small ruminants, followed by cattle (3.8%) and the lowest (2.8%) was seen in pigs (Table 2). The highest number of false positive reactions was observed in pigs (*n* = 31), followed by cattle (*n* = 23) and small ruminants (*n* = 6).

**Table 2:** Seroprevalence of anti-*Brucella* antibodies by species at slaughter in three regions of Uganda**.**

Species	Number of positive sera		Prevalence (%)		95%CI	FPSR
	RBT	NH-Immunoprecipitation test (n)	AP (n/N)	TP		
Cattle (*N* = 886)	54	31	3.5	3.8	2.4–4.9	23
Small ruminants (*N* = 925)	57	51	6.2	6.7	4.2–7.1	6
Swine (*N* = 900)	48	17	1.9	2.8	1.1–2.9	31

### Relationship between epidemiological factors and anti-*Brucella* seropositivity

On univariable analysis a significant association was observed between the origin of slaughter cattle and their serostatus (*p* = 0.045) (Table 3). Similarly, a significant association was observed between the sex of small ruminants (*p* < 0.001), the breed (*p* < 0.001), their origin (*p* < 0.001) and their anti-*Brucella* serostatus. No significant association was observed between the age, sex, breed, origin of slaughtered pigs and their serostatus.

**Table 3:** Univariable analysis of relationships between different epidemiological factors and anti-*Brucella* seropositivity in different species.

Variable	Positive/Total (%)	95%CI	Total/n (%)	X^2^	*p*-value
**Cattle (*n* = 886)**					
Age					0.358
Adult	30/795 (3.8)	2.6–5.3	795/886 (89.7)	–	
Juvenile	1/91 (1.1)	0.1–5	91/886 (10.3)		
Sex				1.25	0.264
Male	14/501 (2.8)	1.6–4.5	501/886 (56.5)		
Female	17/385 (4.4)	2.7–6.8	385/886 (43.5)		
Breed				–	0.144
Local	29/736 (3.9)	2.7–5.5	736/886 (83.1)		
Cross	2/150 (1.3)	0.3–4.2	150/886 (16.9)		
Origin				–	0.045*
Central	14/401 (3.5)	2.0–5.6	401/886 (45.3)		
Northern	4/257 (1.6)	0.5–3.7	257/886 (29)		
Eastern	13/228 (5.7)	3.2–9.3	228/886 (25.7)		
**Small ruminants (*n* = 925)**					
Age				–	0.052
Adult	49/807 (6.1)	4.6–7.9	807/925 (87.2)		
Juvenile	2/118 (1.7)	0.4–5.3	118/925 (12.8)		
Sex				16.76	<0.001**
Male	10/448 (2.2)	1.2–3.9	448/925 (48.4)		
Female	41/447 (8.6)	6.3–11.4	477/925 (51.6)		
Breed				12.69	<0.001**
Local	34/786 (4.3)	3.1–5.9	786/925 (85)		
Cross	17/139 (12.2)	7.6–18.4	139 /925 (15)		
Origin				–	<0.001**
Central	34/356 (9.6)	6.8–12.9	356/925 (38.5)		
North	0/325 (0)	0–0.8	325/925 (35.1)		
East	17/224 (7)	4.3–10.7	244/925 (26.4)		
Species				2.41	0.13
Caprine	45/727 (6.19)	4.6–8.1	727/925 (78.6)		
Ovine	6/198 (3.03)	1.3–6.1	198/925 (21.4)		
**Pigs (*n* = 900)**					
Age				–	0.143
Adult	13/524 (2.5)	1.4–4.1	524/900 (58.2)		
Juvenile	4/376 (1.1)	0.4–2.5	376/900 (41.8)		
Sex				2.52	0.112
Male	11/386 (2.8)	1.5–4.9	386/900 (42.9)		
Female	6/514 (1.2)	0.5–2.4	514/900 (57.1)		
Breed					>0.9
Local	6/297 (2.0)	0.8–4.1	297/900 (33.0)		
Cross	11/603 (1.8)	1–3.1	603/900 (67.0)		
Origin				1.62	0.445
Central	10/589 (1.7)	0.9–3	589/900 (65.4)		
Northern	6/210 (2.9)	1.2–5.8	210/900 (23.3)		
Eastern	1/101 (1)	0.1–4.5	101/900 (11.2)		

* Significant predictors of anti-*Brucella* seropositivity at *p* < 0.05.

** Significant predictors of anti-*Brucella* seropositivity at *p* < 0.01.

### Multivariable risk factor analysis for anti-*Brucella* antibody seropositivity

Cattle originating from the Eastern districts were associated with increased odds (OR = 4.65,95%CI = 1.49–17.75, *p* = 0.013) of being seropositive for anti-*Brucella* antibodies compared to those from the Northern districts (Table 4). Indigenous cattle were associated with increased odds (OR = 3.74, 95%CI =1.09–23.46) of being seropositive for anti-*Brucella* antibodies compared to cross breed cattle although the relationship was not significant (*p* = 0.074). Female cattle were associated with slightly increased odds (OR = 1.09, 95%CI = 0.49–2.43) of being seropositive for anti-*Brucella* antibodies compared to male cattle although this was not significant (*p* = 0.829). The model was evaluated using the Hosmer-Lemeshow goodness of fit with backward selection procedure (X^2 ^= 6.524, *p* = 0.258).

**Table 4:** Factors associated with anti-*Brucella* sero status.

Variable (Reference category)	OR	95% CI	*p*-value
**Cattle (HL goodness of fit, *X^2^ = 6.524, p = 0.258)***			
Breed (Cross)			
Indigenous	3.74	1.09–23.46	0.074
Origin (North)			
Eastern	4.65	1.49–17.75	0.013*
Central	2.96	1.03–10.67	0.062
Age (Juvenile)			
Adult	3.44	0.71–62.07	0.23
Sex (Male)			
Female	1.09	0.49–2.43	0.829
**Small ruminants (HL goodness of fit, *X^2^ = 5.33, p = 0.62)***			
Age (Juvenile)			
Adult	4.04	1.16–25.5	0.006**
Sex (Male)			
Female	2.93	1.46–6.42	0.004**
Breed (Indigenous)			
Cross	1.08	0.51–2.33	0.833
Origin (Central)			
Eastern	0.64	0.31–1.32	0.22
Northern			0.98
Species (Sheep)			
Goats	2.53	1.08–6.98	0.048*
**Pigs (HL goodness of fit, *X^2 ^= 6.5538, p = 0.4768)***			
Age (Juvenile)			
Adult	2.5	0.81–9.38	0.131
Sex (Male)			
Female	2.88	1.07–8.52	0.041*
Breed (Cross)			
Local	1.32	0.42–3.75	0.617
Origin (Eastern)			
Northern	3.55	0.49–72.75	0.274
Central	2.47	0.42–47.23	0.408

* Significant predictors of anti-*Brucella* seropositivity at *p* < 0.05.

** Significant predictors of anti-*Brucella* seropositivity at *p* < 0.01.

Adult small ruminants were associated with increased odds of being sero-positive compared to juveniles (OR = 4.04, 95%CI = 1.16–25.5, *p* = 0.006). Female small ruminants were more likely to be seropositive for anti-*Brucella* antibodies (OR = 2.93, 95%CI = 1.46–6.42, *p* = 0.004) compared to males. Among the small ruminants, goats had increased odds of being seropositive for anti-*Brucella* antibodies compared to sheep (OR = 2.53, 95%CI = 1.08–6.98, *p* = 0.048). The model was evaluated using Hosmer-Lemeshow goodness of fit with backward selection procedure (X^2^ = 5.33, p = 0.62). Similarly, female pigs were more likely to be seropositive (OR = 2.88, 95%CI = 1.07–8.52, *p* = 0.041) for anti-*Brucella* antibodies compared to male pigs. The model was evaluated using the Hosmer-Lemeshow goodness of fit test with the back ward lection procedure (X^2 ^= 6.5538, *p* = 0.4768).

## Discussion

Despite the shortcomings of serological tests, serological surveillance remains the cornerstone of brucellosis control efforts in resource limited settings in sub-Saharan Africa (30). Slaughterhouses are sentinel sites and form a vital component of a brucellosis surveillance systems. Livestock from different epidemiological contexts converge here and can be easily sampled to monitor exposure to *Brucella* species*.* Our findings show that the buying and selling of slaughter cattle and small ruminants is more widespread across the country compared to pigs (Figure 1). This therefore underscores the importance of cattle and small ruminants as a major source of livelihoods to farmers, the traders, other value chain actors and as a major revenue source for the respective sub national governments (31). However, the importance of pigs in rendering similar benefits should not be underestimated. Given that the movement of cattle and small ruminants is more widespread compared to pigs, their potential in dissemination of *Brucella* species from one region to another is high. Movement of cattle and small ruminants for trade and search of water and pastures has been documented as a main factor in spread of brucellosis in other African countries such as Mali and Côte d’Ivoire (32). The Central part of Uganda represents the largest meat consuming population and hence the highest number of livestock slaughtered and sampled in this region relative to others (Table 1).

Serological testing is a vital component of brucellosis interventions in endemic countries including prevalence estimation, assessment of efficacy of control and elimination measures or confirmation of freedom from the disease (10, 33). Detection of antibodies to *Brucella* form the basis for indirect diagnosis especially for large scale surveillance purposes (34, 35). For more than 2 decades serological tests have formed the core of most of the studies involving brucellosis in Uganda, due to their affordability, availability, and low technological requirements. Available literature indicates that in the 2010–2019 decade the commonly used serological tests for animal brucellosis in Uganda included RBT, SAT, i/c-ELISA, FPA and CFT (30, 36). Each of these tests has advantages and shortcomings regarding brucellosis serology.

In the current study, we employed a serial testing strategy using a combination of the RBT and the NH immunoprecipitation test to provide brucellosis sero-prevalence with high specificity, ruling out the FPSR and the interference that the unknown vaccination status of ruminants presents in Uganda. In NH immunoprecipitation tests, a positive reaction requires a threshold combination of antibody titers and avidity resulting from active *Brucella* infection, which threshold cannot be reached during milder and transient infections caused by vaccination or extracellular bacteria associated with FPSR (8). Thus, even though the DSe is not as high as those of RBT or iELISA, particularly in the case of pig sera, the very high DSp of the method renders the results unequivocal with regards to infection. This approach gives improved accuracy over the more commonly used methods. For example, the SAT test detects IgM and agglutinating IgG antibodies directed against *Brucella* but fails to detect non-agglutinating IgG (8). Thus, SAT is not recommended by the OIE for animal brucellosis. Both the RBT and appropriately validated i-ELISAs show optimal DSe and DSp in the absence of vaccination and are recommended for detecting infected herds or to demonstrate absence of infection in brucellosis-free herds (9). However, these tests cannot differentiate between natural infection and vaccination with S19 or Rev1 vaccines. The CFT shows slightly less DSe and its DSp is also compromised in the presence of S19 vaccination or due to FPSR. The c-ELISA reduces but does not eliminate residual antibody produced in response to vaccination with S vaccines. The improved specificity of the c-ELISA parallels the reduction in sensitivity compared to i-ELISA (9). It is therefore necessary in future studies to investigate positive reactions with complementary tests taking into account the epidemiological context (9).

The seroprevalence of anti-*Brucella* antibodies was highest in small ruminants (6.7%), followed by cattle (3.8%) and lowest in pigs (2.8%) (Table 2). Kakooza found similar seroprevalence rates during a sero-monitoring survey of slaughtered goats and sheep in Kampala using a combination of RBT (6.3%) and i-ELISA (6.6%) (37). Our findings are also consistent with those of Miller, who reported a high seroprevalence of 17% in goats during their study on brucellosis on farms in South Western Uganda using the RBT (22). In our study the observed seroprevalence in cattle is lower than the 14% previously observed by (22) who used RBT. However, using commercial c-ELISA Makita reported 5% prevalence of anti-*Brucella* antibodies in cattle in urban areas of Kampala which is slightly higher than that observed in our study (38). The results of the above previous studies should however be interpreted considering the potential effect of both FPSR and post vaccination interference. Notwithstanding the shortcomings of the tests and the testing strategies employed, these studies clearly indicate a higher prevalence of brucellosis in small ruminants compared to cattle. The findings of our study reaffirm these earlier observations. The high prevalence of anti-*Brucella* antibodies in small ruminants imply that these species are very important in the maintenance and dissemination of *Brucella* species with prevention and control implications especially in mixed grazing systems. In addition, due to their small size, movement restrictions are harder to enforce by state veterinary authorities leading to dissemination of disease across wide geographical areas. A high number of FPSR was observed in cattle (*n* = 23) (Table 2). However, these could not be attributed to vaccination because none of these indicated bands consistent with vaccination in the NH immunoprecipitation test. Besides, there is very scanty information regarding ruminant vaccination with S vaccines in Uganda. Therefore, in the absence of this information about vaccination, the FPSR could be attributed to cross reacting bacteria.

Available literature provides conflicting accounts of the possible involvement of pigs in the epidemiological cycle of brucellosis in Uganda. The earliest account was given by Mwebe who reported a 9% seroprevalence of *Brucella* antibodies in swine sera previously collected and bio-banked in veterinary diagnostic laboratories in Uganda from 1998 to 2008 although there is no mention of the serological test used to reach these conclusions (24). Later using real-time PCR Atherstone found no evidence of *Brucella* in pigs slaughtered in a major slaughterhouse in Kampala but molecular methods remain to be validated for the diagnosis of this infection in pigs (39). More recently Erume, using a combination of i-ELISA and CFT reported a sero-prevalence of less than 1% in their study of pigs sampled from the district of Kamuli in Eastern Uganda, Masaka and Mukono in Central Uganda (16). The large number of negative results in this study could have several explanations one of them being that the commercial kits often used in these studies are not validated for use in pigs under Uganda conditions. By using a highly specific test based on detection of antibodies in swine sera, the observed sero-prevalence of 2.8% in this study represents a slight departure from the findings of the earlier studies. Previously, Akoko also detected anti-*Brucella* antibodies and DNA in swine serum collected from slaughterhouse pigs confirming the presence of *Brucella* in pigs in Kenya (40). Nevertheless, the role of pigs in the epidemiological cycle of brucellosis in Uganda seems to be minor, covert, and not clearly understood. The emerging practice of feeding ruminant viscera (offcuts) to pigs in some parts of the country may be a potential source of infection to pigs and needs to be investigated since pigs may occasionally be infected with *B. abortus* in endemic areas (6).

Cattle from the Eastern part of the country were more associated with *Brucella* sero positivity compared to those from other parts of the country (Tables 3, 4). A large part of this region especially the Karamoja sub-region is hard to reach and qualified veterinary services required for implementing control strategies are still inadequate (41). Moreover, communal grazing is still largely practiced due to communal land ownership. Communal grazing practices and the transhumant movement in search of water and pastures facilitates mixing of herds and dissemination of *Brucella* species (21)*.* Therefore, risk based or targeted interventions should be considered in future strategies aimed at progressive control of brucellosis in Uganda.

The greater likelihood of female small ruminants being more seropositive compared to males is probably related to the role of females in amplification and dissemination of *Brucella* species from the udder and their pregnant uterus that act as a good medium for their establishment (Tables 3, 4). The higher concentration of erythritol in the gravid uterus provides a suitable medium for the proliferation of *Brucella* (42). The resultant placentitis results into abortion and concomitant shedding of *Brucella* via aborted fetus and fetal fluids that contaminate the environment thereby presenting a risk for infection of susceptible animals (42). Moreover, female small ruminants are likely to stay longer in the flocks for breeding purposes there by increasing the chance of being exposed to the bacteria.

The fact that *Brucella* is silent in young animals perhaps accounts for the higher likelihood of seropositivity observed in adult small ruminants in this study (Table 4) (9). In addition, the high concentration of erythritol that facilitates the growth of *Brucella* species in mature uteri further explains the age-related difference in seropositivity (9). In their study of small ruminant brucellosis in Karenga district in north eastern Uganda, Akwongo and Kakooza found that goats were more exposed to brucellosis than sheep (43). Similarly in the current study, goats were more likely to be seropositive compared to sheep. This difference, however, could be attributed to sampling frame. In this study, more goats were slaughtered compared to sheep resulting in a smaller number of sheep having a chance of being sampled. It is difficult to explain why none of the small ruminants sampled from northern Uganda had anti-*Brucella* antibodies. However, a combination of factors could account for this. First the northern region has the lowest number of cattle and sheep and therefore less grazing pressure (44). The likelihood of small ruminants getting in contact with contaminated pasture is therefore less. Moreover, due to the preference for browse, the chances of goats grazing on contaminated pasture is reduced in preference for the widely abundant browse.

Although literature suggests that *B. suis* affects both sexes equally (6), the higher likelihood of seropositivity in females may also be related to their role in amplification and dissemination of *Brucella,* and to flock structure where more female pigs are kept compared to males for breeding purposes. *B. suis* infection in pigs is more generalized and characterized by persistence of the organism in the uterus leading to chronic metritis, abortions, still births in female pigs and release of *Brucella* in vaginal discharges that further cause environmental contamination (42).

Despite the epidemiological information regarding the seroprevalence of brucellosis in sheep and goats in Uganda, control in these animals is largely neglected. The apparent neglect is related to lack of awareness of state veterinary authorities and farmers on the magnitude of the problem and the obvious possibility of cross-infections, and because small ruminants are perceived of lower value in terms of income compared to cattle. Our study has demonstrated that slaughterhouse surveillance is a viable avenue for monitoring brucellosis and the findings can be used as a basis for targeted control.

Slaughterhouses in Uganda are not well organized and regulated, with very few centralized slaughter facilities in the study area. This means that animals slaughtered in other areas other than the main slaughterhouses could not be sampled thereby reducing the sampling frame. Since sampling was not done at farm level, we entirely relied on the person presenting the animal for slaughter to declare the origin of the animal. Therefore, the information provided regarding the origin of the livestock may not be entirely accurate. The livestock population at slaughter may not be representative of the livestock population as there are different reasons that animals are sold for slaughter such as infertility, illness, old age. This needs to be considered when generalizing the results to the broader population.

## Conclusions and recommendations

Brucellosis remains a huge animal production challenge in Uganda with small ruminants most affected followed by cattle and pigs. The findings indicate that pigs may be playing a minor and covert role in its epidemiology. Regional differences in *Brucella* seropositivity have been detected with the eastern part of the country most affected regarding cattle brucellosis. However, considering that only animals presented for slaughter at centralized slaughter facilities were sampled and tested, the sero-prevalence in the national herd could be higher. Consequently, the risk to slaughterhouse workers, other meat handlers and consumers is potentially high.

We recommend that although cattle are essential in sustaining livelihoods and are usually the central focus of animal disease control programs, future interventions to control brucellosis in Uganda should include sheep and goats since they play a critical role in the maintenance and dissemination of *Brucella.* Comprehensive bacteriological and molecular studies with a wider geographical focus should be undertaken to clarify small ruminants to cattle transmission and the role of pigs in the epidemiological cycle of brucellosis in Uganda.

## Data Availability

The raw data supporting the conclusions of this article will be made available by the authors, without undue reservation.
